# Reconstructing Indian-Australian phylogenetic link

**DOI:** 10.1186/1471-2148-9-173

**Published:** 2009-07-22

**Authors:** Satish Kumar, Rajasekhara Reddy Ravuri, Padmaja Koneru, BP Urade, BN Sarkar, A Chandrasekar, VR Rao

**Affiliations:** 1 Anthropological Survey of India, 27 Jawaharlal Nehru Road, Kolkata 700 016, India; 2 Department of Genetics, Southwest Foundation for Biomedical Research, San Antonio, TX 78227, USA

## Abstract

**Background:**

An early dispersal of biologically and behaviorally modern humans from their African origins to Australia, by at least 45 thousand years via southern Asia has been suggested by studies based on morphology, archaeology and genetics. However, mtDNA lineages sampled so far from south Asia, eastern Asia and Australasia show non-overlapping distributions of haplogroups within pan Eurasian M and N macrohaplogroups. Likewise, support from the archaeology is still ambiguous.

**Results:**

In our completely sequenced 966-mitochondrial genomes from 26 relic tribes of India, we have identified seven genomes, which share two synonymous polymorphisms with the M42 haplogroup, which is specific to Australian Aborigines.

**Conclusion:**

Our results showing a shared mtDNA lineage between Indians and Australian Aborigines provides direct genetic evidence of an early colonization of Australia through south Asia, following the "southern route".

## Background

The greatest ever reconstructed journey of our own species (*Homo sapiens*) begins in Africa with a group of hunter-gatherers, perhaps just a few hundred strong and ends some 150 – 200 thousand years (ky) later with their six and a half billion descendants spread across the occupied world. Most of the DNA and archaeological evidence are in agreement of the proposition. However, route(s) and time of such spread, undertaken by the anatomically modern Africans to populate the world has been the greater untold part of the story. Recent genetic studies (especially those based on mitochondrial DNA) suggest single "southern route" dispersal of modern humans, extended from the Horn of Africa, across the mouth of the Red Sea into Arabia and southern Asia some time before 50 ky [1-7]. Subsequently, the modern human populations expanded rapidly along the coastlines of southern Asia, southeastern Asia and Indonesia to arrive in Australia at least by 45 thousand years before present (kyBP), best represented by the anatomically modern human skeleton from the site of Lake Mungo 3 in New South Wales [1,8-16]. An early phylogenetic link between Indians and Australian Aborigines has also been suggested by observations based on morphology [17]. The major challenge to this scenario is to document individual steps in this colonization process based on genetics and archaeological evidence. The mtDNA lineages sampled so far from south Asia, eastern Asia and Australasia show non-overlapping distributions of haplogroups within macrohaplogroups M and N and its subclade R [10]. The archaeological map of Arabia and India are at present largely blank for the critical period from ~50 to ~60 kyBP [18,19]and whatever intriguing hints of early modern human occupations are available from the site of Patne in western India, [20]Jwalapuram in southern India [21] and Batadomba-lena in Sri Lanka [22,23] suggest closer affinities to African Middle Stone Age traditions, [3,21] whereas, similarly "advanced" technologies in the area to the east of the Indian subcontinent, especially in the relatively well-explored area of Australia and New Guinea are lacking [3,8,11].

## Results and discussion

The complete mtDNA sequencing indicate that both Australians and New Guineans exclusively belongs to the out-of-Africa founder types M and N, thus ultimately descended from the same African emigrants ~50 to 70 kyBP, as all other Eurasians [24]. However, in context of the Eurasian phylogeny [25-35], shared branches more recent than the founding types M, N, and R have not been reported so far, except a shared variant at nucleotide position 8793 between Australian specific haplogroup M42 and East/Southeast Eurasian specific haplogroup M10 [24].

**Figure 1:**
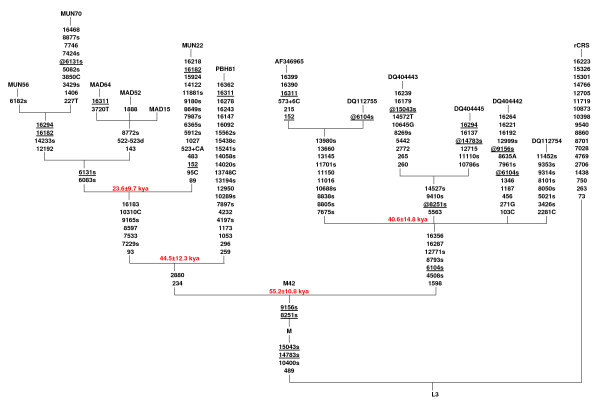
**Phylogenetic reconstruction of M42 Lineage**. The phylogenetic reconstruction was performed using 7 New mtDNA Sequences from India and 6 Australian Aborigines mtDNA sequences from published source [2,25,36]. The sequence region np 16024 to 434 is missing in two (i.e. DQ112754 and DQ112755) published sequences. Suffixes A, C, G, and T indicate transversions, "d" signifies a deletion and a plus sign (+) an insertion; "s" indicates synonymous polymorphisms; recurrent mutations are underlined. The prefix "@" indicates back mutation. The coalescence age estimates calculated as per Kivisild et al [2] are presented in thousand years ago (kya). Variation at hypervariable positions 16184–16193, 16519 and insertion C at 309 and 315 are not shown.

Our complete mtDNA sequencing of 966 individuals from 26 relic populations of India identified seven individuals from central Dravidian and Austro-Asiatic tribes who share two basal synonymous mtDNA polymorphisms G8251A and A9156T with the M42 haplogroup, which is specific to Australian Aborigines. The phylogenetic reconstruction of 7 Indian (this study) and 6 Australian Aborigine mtDNA sequences from published source [2,25,36] is shown in Figure 1, and it differs from the previous reports [24,36] in the placement of the G8251A polymorphism. This polymorphism together with A9156T is present in all 7 Indian samples of this study, as well in one Indian sample (i.e. PU202) reported previously based on RFLP [37-39] and in 4 out of 6 Australian sequences used in this reconstruction. Both G8251A and A9156T are considered ancestral to M42, but the lack of G8251A in an Australian sub lineage consisting of two genomes indicates a back mutation event. Being based on the combination of two synonymous polymorphisms and their replication in quite a few Indian samples (7 in this study and one reported previously [37]), the present phylogenetic reconstruction of the haplogroup M42 seems parsimonious and more stable than the previously suggested M10 and M42 link through 8793 polymorphism [24].

The coalescence time estimate 55.2 ± 10.8 kyBP of the average sequence divergence of the Indian and Australian M42 coding-region sequences from the root is consistent with the first evidence of human occupation provided by 11 silcrete flakes with plain and relatively thick striking platforms recovered from below the lowest gravels in the barrier sands of the Mungo B trench, [40] bracketed by ages of 50.1 ± 2.4 and 45.7 ± 2.3 kyBP [8]. The similar or slightly older ages for the initial human arrival in northern and western Australia [41-43] also seem to be in agreement. The underlying deposits at Mungo B trench, dated to 52.4 ± 3.1 kyBP, appear to be culturally sterile [8]suggesting colonization of continental Australia some time after 50 kyBP from south Asia.

The shared lineage provides direct genetic evidence to the long suggested ancient link between India and Australia [17,44,45]. However the deep divergence (i.e. 55.2 ± 10.8 kyBP) of the Indian and Australian branches within M42, coupled with the evidence of the earliest and most pronounced population expansion outside Africa in Southern Asia estimated to ~52 kyBP using Bayesian Skyline analysis [46] followed by high mtDNA diversity in Indian populations [2,4,10,15,27,33], strongly suggest that Australia perhaps along with East/Southeast Eurasia and Papua New Guinea [24] was populated from Southern Asia plausibly slightly before or in the beginning of the population expansion that has given rise to a large number of mtDNA lineages within macrohaplogroup 'M' in India.

## Conclusion

Our results showing a shared mtDNA lineage between Indians and Australian Aborigines provides direct genetic evidence that Australia was populated by modern humans through south Asia following the "Southern Route". The divergence of the Indian and Australian M42 coding-region sequences suggests an early colonization of Australia, ~60 to 50 kyBP, quite in agreement with archaeological evidences.

## Methods

With the above background, a total of 966 mitochondrial DNAs (mtDNAs) were completely sequenced from 26 relic tribes of India. Each sample comprises unrelated healthy donors from whom appropriate informed consent was obtained. The ethical clearance for the study was obtained from the organizational ethical clearance committee of Anthropological Survey of India.

DNA was extracted from all the collected 4–5 ml blood samples using standard phenol-chloroform methods [47] with minor modifications. For complete mtDNA sequencing, DNA was PCR amplified following standard protocols and using the PCR primers and conditions of Rieder *et al*. [48]. PCR product was sequenced with both forward and reverse primers using BigDye Terminator v3.1 sequencing kits from Applied Biosystems on an Applied Biosystems 3730 automated DNA analyzer. Contig assembly and sequence alignments were accomplished with SeqScape v2.5 software from Applied Biosystems. Mutations were scored relative to the revised Cambridge Reference Sequence (rCRS) [49] with each deviation confirmed by manual checking of electropherograms. The phylogenetic tree was reconstructed from median-joining networks rooted to L3 using NETWORK 4.2.0.1 software [50]. The tree was checked manually to resolve homoplasies. To confirm the strength of the present phylogenetic reconstruction, we also searched the complete mtDNA sequences available at [51], [52] and [53] for the mitochondrial genome(s) harboring polymorphisms G8251A and A9156T and no similar sequence other than those used in the present phylogenetic reconstruction were found. The coalescent age estimates were calculated by Rho (**ρ**) statistics [54] and using mutation rate of one synonymous transition per 6,764 years [2] calibrated on the basis of an assumed human-chimp split of 6.5 million years ago. Standard errors for coalescence estimates were calculated following Saillard et al [54]. The seven new complete mtDNA sequences used in the Phylogenetic reconstruction in this study have been submitted to GenBank (accession numbers FJ380210–FJ380216). Our other complete mtDNA sequences under publication elsewhere can also be found at GenBank with accession numbers FJ383174–FJ383814.

## Abbreviations

ky: Kilo Years; kyBP: Kilo Years Before Present: mtDNA: Mitochondrial DNA; rCRS: Revised Cambridge Reference Sequence; np: Nucleotide Position; PCR: Polymerase Chain Reaction.

## Authors' contributions

SK, RRR and PK carried out initial screening and complete mtDNA sequencing of the data. SK and RRR did sequence alignment, data base search and all the phylogenetic analysis. AC, BNS and BPU contributed samples. SK drafted the manuscript. VRR conceived the study, participated in its design and coordination also helped to improve the manuscript. All authors read and approved the final manuscript.
